# The Effects of the Microbial Biostimulants Approved by EU Regulation 2019/1009 on Yield and Quality of Vegetable Crops

**DOI:** 10.3390/foods11172656

**Published:** 2022-09-01

**Authors:** Giovanna Marta Fusco, Rosalinda Nicastro, Youssef Rouphael, Petronia Carillo

**Affiliations:** 1 Department of Environmental, Biological and Pharmaceutical Sciences and Technologies, University of Campania “Luigi Vanvitelli”, Via Vivaldi 43, 81100 Caserta, Italy; 2 Department of Agricultural Sciences, University of Naples Federico II, 80055 Portici, Italy

**Keywords:** arbuscular mycorrhizal fungi (AMF), *Azospirillum*, *Azotobacter*, *Rhizobium*, nutritional quality, plant growth-promoting bacteria, PRISMA method

## Abstract

The use of microbial biostimulants such as plant growth-promoting rhizobacteria (PGPB) and arbuscular mycorrhizal fungi (AMF) has gained popularity in recent years as a sustainable approach to boost yield as well as the quality of produce. The beneficial effects of microbial biostimulants have been reported numerous times. However, information is missing concerning quantitative assessment of the overall impact of microbial biostimulants on the yield and quality of vegetable crops. Here we provide for the first time a comprehensive, semi-systematic review of the effects of microbial biostimulants allowed by Regulation (EU) 2019/1009, including microorganisms belonging to the AMF (phylum Glomeromycota), or to *Azospirillum*, *Azotobacter* and *Rhizobium* genera, on vegetable crops’ quality and yield, with rigorous inclusion and exclusion criteria based on the PRISMA method. We identified, selected and critically evaluated all the relevant research studies from 2010 onward in order to provide a critical appraisal of the most recent findings related to these EU-allowed microbial biostimulants and their effects on vegetable crops’ quality and yield. Moreover, we highlighted which vegetable crops received more beneficial effects from specific microbial biostimulants and the protocols employed for plant inoculation. Our study is intended to draw more attention from the scientific community to this important instrument to produce nutrient-dense vegetables in a sustainable manner. Finally, our semi-systematic review provides important microbial biostimulant application guidelines and gives extension specialists and vegetable growers insights into achieving an additional benefit from microbial biostimulant application.

## 1. Introduction

Over the last century, the global population has more than quadrupled, increasing from about 1.91 billion in 1922 [1] to 7.96 billion as of July 2022, as reported by the most recent United Nations estimates [2]. Moreover, in 2017 AsiaNews, an official press agency of the Roman Catholic Pontifical Institute for Foreign Missions, revealed the existence of more than a billion people around the world (mainly in Asia and Africa), one-third of them children, not registered by their national governments and therefore with no identities and no rights [3]. Therefore, the current world population may have already crossed the 9 billion mark or will do so between late 2022 and early 2023, with the demand for food already far exceeding current production. In these conditions, in which sustainable development looks hardly achievable, it is of pivotal importance to diversify the energy mix and raw material sourcing in Europe [4] while trying to reduce energy demand and the use of synthetic fertilizers. High-input, resource-intensive farming systems, like horticultural greenhouses, entail the highest use of chemicals and direct and indirect pollution in agriculture [5,6]; however, is it possible to reduce fertilizer use without sacrificing food production and quality? The solution for meeting the demand for food by providing healthy and nutritious products to a growing population while resources are becoming increasingly scarce and without further contributing to climate change and pollution appears extremely complicated. However, a promising strategy that could open up a more environmentally friendly horticulture paradigm, reducing the use of synthetic fertilizers while increasing the resource use efficiency (RUE), quality and yields of agricultural produce concerns the use of plant biostimulants (PBs) [7,8,9,10]. The European Biostimulants Industry Council (EBIC) fosters the use of PBs, helping farmers to profitably grow adequate quantities of high-quality agricultural food while using resources wisely. According to the EBIC, PBs are made of substances (plant and seaweed extracts, protein hydrolysates, mineral salts and humic acids) and/or microorganisms that, when applied in low quantities to plants or the rhizosphere, can enhance one or more crop plant characteristics such as nutrient uptake, RUE, abiotic stress tolerance and quality traits [8]. The beneficial interactions with arbuscular mycorrhizal fungi (AMF) or plant growth-promoting bacteria (PGPB), which for the most part are nitrogen-fixing symbiotic bacteria, may improve crop plants’ growth and development, enhancing the availability and/or uptake and assimilation of nutrients, even under suboptimal nutrient conditions and/or abiotic stresses [11,12]. These microorganisms, which have co-evolved with their hosts, have been shown able to increase crop yields and tolerance to abiotic stress by co-ordinately regulating primary and secondary metabolism pathways in plants [13,14,15,16]. This may happen because their cells are much more numerous than those of plants, and their genome is also commonly defined as the second genome of the plant or its microbiome [17]. These microorganisms interact positively with the plant in the rhizosphere, enhancing nutrient availability in the soil, nutrient uptake and nutrient assimilation [18]. In fact, they are not only able to carry out atmospheric nitrogen fixation (specifically PGPB) and synthesis of metabolites like amides or phytohormones exportable to plants, but they can play a role in the decomposition of organic matter, solubilization of insoluble P-containing minerals and uptake of water, nutrients and trace elements and their delivery into the roots [19]. The ability to produce antimicrobials allows them to antagonize the action of plant pathogens by indirectly defending the plant [20]. Of particular interest are mycorrhizal fungi, which establish symbiosis with over 90% of plant species, and among the main forms of physical interaction, the most common is the formation of arbuscules (AMF). This type of association leads to an increase in nutritional efficiency, especially for the exchange of phosphate [21], ammonium, nitrate, calcium, zinc and iron in conditions of nutrient deficiency or low availability. In fact, the external hyphae constitute a dense network that increases the surface responsible for the uptake of nutrients and the secretion of organic metabolites capable of binding and/or solubilizing nutrients [22]. Moreover, they have the ability to increase the tolerance mechanisms of plants against abiotic stresses by inducing a greater accumulation in the cell wall of exopolysaccharides, protein-lipopolysaccharides and lipid-polysaccharides, which create a protective biofilm layer on the root surface. Furthermore, by accumulating in their cells, the solutes present in the surrounding solution reduce their toxic concentration. In this way the plants are able to improve water retention even under salinity, resulting in them being more tolerant to this stress condition [23]. Recent studies demonstrated that the network of fungal hyphae constitutes not only a fungus–plant interaction system, but also a connection and signalling system among plants that occupy the same area [24].

Bacteria with potential biostimulant actions belong to different genera, such as *Rhizobium*, *Bradyrhizobium*, *Azotobacter*, *Azospirillum*, *Pseudomonas* and *Bacillus* [25], however only three of them, together with AMF, have been approved as PBs by the Regulation (EU) 2019/1009 [26,27,28], which has laid down rules for the production and marketing of fertilizing products within the EU market beginning 16 July 2022. In fact, according to this regulation, an EU fertilizing product may contain “micro-organisms, including dead or empty-cell micro-organisms and non-harmful residual elements of the media on which they were produced, which have undergone no other processing than drying or freeze-drying”, but only those belonging to mycorrhizal fungi (AMF, phylum Glomeromycota) or to the *Azospirillum*, *Azotobacter* or *Rhizobium* genera. These latter microorganisms were chosen on the basis of their phenotypic characteristics, and in particular their ability to fix atmospheric nitrogen or to solubilize phosphorus compounds. However, in the last two decades, new sequencing techniques have allowed radical changes in these obsolete classifications, prompting the need for an immediate review of the list of candidates for biostimulants in the EU Regulation 2019/1009, as recently evidenced by Hendriksen [28].

That said, and considering the limits previously highlighted, a comprehensive review of the effects of microbes allowed by Regulation (EU) 2019/1009 on vegetable crops’ quality and yield, with rigorous inclusion and exclusion criteria based on the PRISMA method, has never done before. Moreover, it has not yet been proven which vegetable crops receive more beneficial effects from microbial biostimulants and in particular from the specific microorganisms the PBs contain. Therefore, we identified, selected and critically evaluated all the relevant research studies from 2010 onward in order to provide a critical appraisal of the most recent studies related to the microbial biostimulants approved by Regulation (EU) 2019/1009 [26] and their effects on vegetable crop quality and yield.

In particular, a list of the species proved to possess biostimulant properties was created and the several protocols employed for plant inoculation presented. This allowed us to highlight the beneficial effects of these PBs on yield and quality of vegetable crops and bring more attention to this important instrument to sustainably address the demand for good-quality food products while helping farmers’ profitability.

## 2. Review Method

A qualitative analysis of peer-reviewed papers on the effects of microbial biostimulants approved by Regulation (EU) 2019/1009 [26] on the yield and quality of vegetable crops was carried out by using the Preferred Reporting Items for Systematic Review and Meta-Analysis (PRISMA) approach [29]. The guidelines of PRISMA allow for the implementation of a selection of published research studies in an unbiased manner, different from other traditional reviews [29]. Moreover, the method to perform traditional reviews very often relies on research articles and review studies already known to review authors or on literature identified using only one scientific database, thus introducing bias. The PRISMA method has been mainly applied to medical studies, but it has proved to be a useful and interesting tool also in the fields of horticulture and food science. Our study shows for the first time the implementation of this systematic review approach to scientific literature related to the effect of selected microbial PBs on horticultural produces.

This review was based on searching the total available records of research in three multidisciplinary abstract databases, namely, PubMed, WoS and Scopus, in order to collect peer-reviewed articles. Boolean operators were used in each database to connect the primary keyword ‘microbial biostimulant’ (or ‘Azotobacter’, ‘Mycorrhiza’/ ‘Mycorrhizal’, ‘Rhizobium’, ‘Azospirillum’) to the subsequent keyword/s ‘food quality’ (and/or ‘nutritional quality’) and ‘yield’ as detailed in Table 1.

By applying thoroughly determined inclusion and exclusion criteria in a systematic search of peer-reviewed articles, potentially relevant articles were collected and evaluated for eligibility.

The search was first narrowed by selecting articles within the subject area of microbial PBs (or name of species or type) that matched also with food quality and/or nutritional quality and/or yield. Articles not in the English language were excluded.

Relevant articles were then selected based on four main criteria: (i) the study must have used a microbial biostimulant and or one of the genera or phylum allowed by Regulation (EU) 2019/1009 [26] in any way related to microbial biostimulants; (ii) the plants or rhizosphere must have been inoculated; (iii) the experimental crop used must have been a vegetable crop; (iv) the study must have evaluated the effect of the PB on edible produce yield and quality. The screening for identifying eligible/relevant studies was performed manually by eliminating duplicates and carefully reading the title, abstract and full text of collected articles in order to assess if they were fully related to the review topic.

## 3. Literature Review

**Figure 1:**
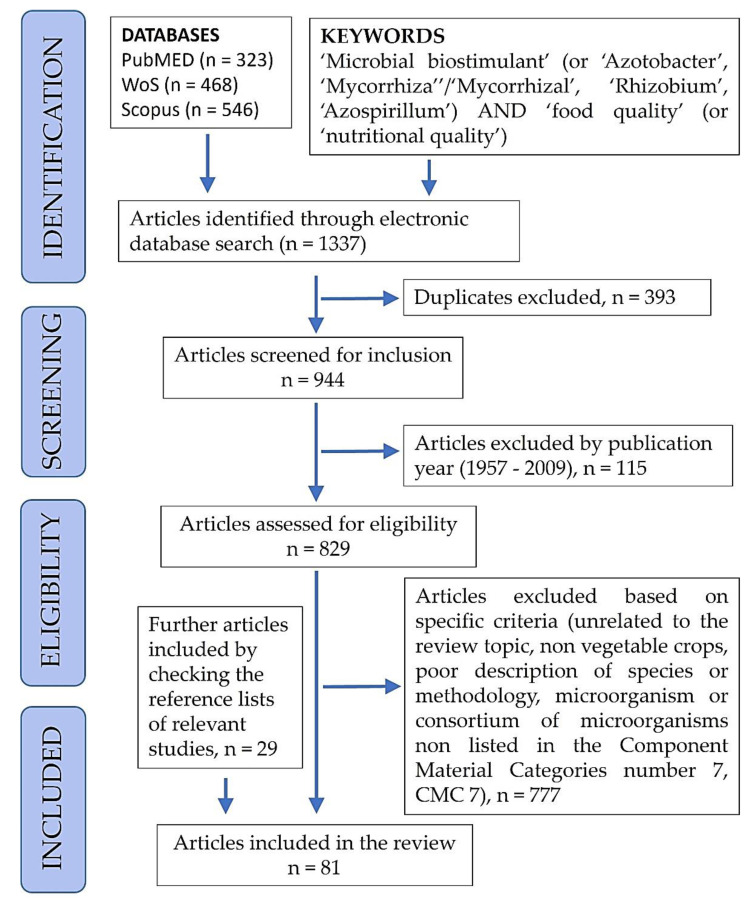
Representation of the PRISMA flow diagram with inclusion and exclusion criteria and corresponding results from the literature search, used to select articles for the review study [29].

In total, we identified 1337 potentially relevant matches through the electronic database search (323 in PubMed, 468 in WoS and 546 in Scopus). We then excluded 393 duplicates, and the remaining 944 articles were screened for eligibility; the complete list in text format of the 944 identified articles is available in Supplemental File S1. Of the identified 944 articles, we firstly eliminated 115 articles published before 2010 because only 2 out of 115 in a time span of 52 years (1957–2009) referred to the effects of allowed microbes on the food quality of vegetable crops [30,31]. Subsequently, we applied screening criteria to eliminate articles unrelated to the review topic, not dealing with vegetable crops, with poor descriptions of species or methodology or dealing with microorganisms or consortia of microorganisms which did not include at least one of the members listed in the Component Material Categories number 7 (CMC 7). We did not exclude review studies as long as they dealt with the topics of the review. The ineligibility screening process eliminated 777 articles and yielded only 52 studies. Another 29 articles were added after checking the reference lists of relevant studies already included, for a final number of articles included in the review equal to 81. Out of the final 81 included articles, 26 were review articles, and 55 were research articles. Figure 1 shows the PRISMA flow diagram for the selection of articles for the systematic review.

## 4. Arbuscular Mycorrhizal Fungi (AMF)

AMF colonization allows the most common symbiotic association between plants and fungi; it is ubiquitous to most natural ecosystems and pivotal to enhancing nutrient use efficiency (NUE) and tolerance to stresses [23]. AMF hyphae, extending from root surfaces to soil areas beyond the zone of mineral resource depletion, improve the absorption and translocation of mineral nutrients [32]. Their inoculation improves the adaptability of host plants to the environment, including that of vegetable crops [33,34], by upregulating nutrient use efficiency and mechanisms for adaptation and tolerance to abiotic stresses like salinity, drought and high or low temperatures [23]. In fact, the majority of vegetable crops can potentially host AMF and obtain benefits from them, even if the efficiency of AMF symbiosis depends on the plant–AMF genotype combination, biotic interactions and environmental conditions [34,35].

In Table 2 are summarized the effects of AMF colonization on vegetable crops, with an overview of the AMF species (in particular using the homotypic synonyms of fungi used in the scientific articles), plant species inoculated, methodology and effects on plants’ quality and yield.

Tran et al. [36], studying the effects of AMF on different plant species, among which were tomato, lettuce, carrot, cucumber, leek and several legumes and cereals, found that plants being differently colonized benefited to varying degrees from symbiosis. In particular, they reported that slow-growing plant species with shorter roots and larger diameters gained more from the symbiotic association with AMF than fast-growing plants with finer and longer roots [36]. In particular, they found that the AMF *Rhizophagus irregularis* (homotypic synonym: *Glomus intraradices*) increased the content of P, Cu, Zn and S in shoots and edible parts (where present) of plants, in particular in leek, whose biomass was also enhanced. The mineral content (N, P, S and Cu) of carrot was also highly increased. Leek and carrot, which are slow-growing and have coarse and large-diameter roots, undergo a higher level of mycorrhizal colonization, gaining more benefits. Therefore, the plant ionome was more affected by the plant species than by inoculation with AMF [36]. Basil inoculation with *Funneliformis mosseae* and *Rhizoglomus irregulare* (homotypic synonyms: *Glomus mosseae* and *Glomus irregulare*, respectively) increased Fe and Mn content [37], decreased the accumulation of the antinutrient nitrate and improved yield [38]. In basil plants under salinity, the same AMF enhanced Na compartmentalization and P availability [37]. *Glomus etunicatum*, *Glomus fasciculatum* (homotypic synonyms: *Claroideoglomus etunicatum* and *Rhizophagus fasciculatus*, respectively) and *Glomus intraradices* increased basil root dry weight, leaf area, plant height, number of lateral branches and mineral content (e.g., N, P, K, Ca, Fe, Cu and Mn) [39]. *G. fasciculatum* inoculation enhanced N, K, S, B, Fe and Zn uptake in basil, and when cultivated with high P also increased biomass and P content [40].

*G. intraradices* [41] and *F. mosseae* and *R. irregulare* [42] increased the mineral content (e.g., N, P and Cu) and growth of the industrial tomato Moneymaker. *F. mosseae* and *R. irregulare* improved the accumulation of Ca and Zn in the yellow cherry tomato Giagiù of the variety “Pomodorino del Piennolo del Vesuvio” [43]; moreover, the same AMF solubilized P sources and improved P uptake and content in cherry tomato (*Solanum lycopersicum* L. var. cerasiforme) [44]. *G. mossae* alone increased the percentage of extra-large fruits in tomato; *G. mossae* together with *T. harzianum* increased total and marketable yield of tomato while decreasing Ca and Mg in fruit tissues, probably because of dilution in larger or more numerous fruits [45].

*Funnelliformis mosseae* and *R. irregulare* increased P, Mg, Fe, Mn and Zn in lettuce even under water deficit; moreover, independently of water availability, these AMF were able to increase plant yield, as well as Ca and Cu content [46]. *G. fasciculatum* or a commercial preparation of *G. intraradices* and *G. mosseae* were also able to increase the mineral content and growth of lettuce [47,48]. *R. intraradices* inoculated in plants under low P concentration improved yield but did not affect Se concentration [49], while *R. intraradices*-based commercial preparation, especially when supplied together with a legume-derived protein hydrolysate (PH), improved lettuce’s fresh marketable yield, dry weight, P, K and Fe via an increase in total root length and surface [50]. AMF commercial inocula and/or *F. mosseae* increased plant growth and macro- and micronutrient use efficiency in cucumber [51]. *G. mossae* was also able to increase the growth and content of P and N in shoots and root tissues of coriander [52]

*G. intraradices* enhanced the content of Mg and K in onion bulb tissues, and S in the presence of saprotrophic fungi [53]. The same AMF plus Se increased garlic and onion bulbs’ yields, as well as their content of P, K and Se, while they increased Mg and microelements (B, Cu, Fe, Mn, Si and Zn) only in onion [54,55].

AMF may improve and increase the defensive capacity of plants by increasing both the amount of antioxidant metabolites—such as phenolic acids, anthocyanins and flavonoids—and the activity of antioxidant enzymes like catalase (CAT) and peroxidase (POD) [56,57,58,59] in addition to phytohormones related to defence signalling [34,60]. This mycorrhiza-induced resistance (MIR), which is unrelated to the improved nutritional status, is quite similar as a mechanism to both pathogen-induced systemic acquired resistance (SAR) and rhizobacterial induced systemic resistance (ISR). In fact, it is associated both with the priming of salicylic acid (SA)-dependent genes (similar to SAR) and, more frequently, with the jasmonic acid (JA)-induced defence response and cell wall defence (similar to ISR) [61]. However, MIR could be not only elicited by AMF, but it could also be the cumulative outcome of a direct interaction between plant–AMF interactions and the plant ISR to other beneficial bacteria present in the mycorrhizosphere [61]. Accordingly, Giovannini et al. [62] have reported that beneficial AMF effects are mainly exerted through a synergistic tripartite association among host plants, mycorrhizal symbionts and bacterial communities living in the mycorrhizosphere. They all together do not contribute only to defence from pathogens, but carry out nitrogen fixation and P solubilization, in addition to the synthesis of phytohormones, siderophores and antibiotics [62,63].

The induced changes in secondary metabolism exerted by AMF are also responsible for the increase in phytochemicals in host plants, which improve plant growth and resilience but not always or in the same way the commercial quality [32]. *Claroideoglomus claroideum* (homotypic synonym: *G. claroideum*) alone was found to increase the total phenols in two artichoke cultivars (Romanesco C3 Italy and Violetto Tema), while, together with *F. mosseae*, it enhanced their antioxidant activity [64]. *G. intraradices* and *G. mosseae* alone, and even more so together, were able to increase the total phenolic content and antioxidant activity of artichoke [65]. Seed priming of two globe artichoke cultivars (Romolo and Istar) with a commercial preparation of *F. mosseae, R. irregulare* and *Trichoderma koningii* determined an increase of total phenols and antioxidant activity and improved primary and total fresh marketable yields [66]. The seed-coating of the same artichoke cultivars with a commercial inoculum containing *R. intraradices, F. mosseae* and *T. atroviride* increased the content of 3-*O*-caffeoylquinic acid, 5-*O*-caffeoylquinic acid and apigenin 7-*O*-glucuronide in primary heads as well as the content of 1,5-di-*O*-caffeoylquinic acid in secondary heads, especially in the Romolo variety [67].

*R. intraradices* enhanced the content of active metabolites (picrocrocin, crocin II and quercitrin) and antioxidant activity in saffron, while a commercial preparation of *F. mosseae* and *R. intraradices* increased saffron flower production and yield [68]. Several AMF species were able to increase total phenolic, pyruvic acid, ascorbic acid, flavonol glucosides and antioxidant enzymes in Iranian onion genotypes, with the greatest beneficial effect caused by *Diversispora versiformis* (homotypic synonym: *Glomus versiforme*) [57]. Inoculation with a commercial preparation containing *F. mosseae* and *R. irregulare* before seeding (highest amount) and colonization at the late-development bulb-growth stage increased quercetin compounds if plants were additionally supplied with ammonium [69]. *G. intraradices* plus Se increased ascorbic acid and flavonoids in onion, and only flavonoids in garlic [54,55]. *R. irregularis* increased vitamin B1, organic acids, photosynthesis, growth and yield in the varieties Karmen, Kuba, Sochaczewska and Wolska of onion [70]. *Glomus mossae* enhanced the essential oil content of coriander [52].

*Funnelliformis mosseae* and *R. irregulare* increased phenolic acids in lettuce independently of water availability and increased isochlorogenic acid content under adequate water supply [46]. However, Avio et al. [71] found that *R. irregulare* more than *F. mosseae* was able to enhance the concentration of phenolics and the antioxidant activity. *G. fasciculatum* or a commercial preparation of *G. intraradices* and *G. mosseae* were also able to increase the content of chlorophylls, carotenoids, ascorbate, anthocyanins, tocopherol and phenols, in addition to starch, soluble sugars and proteins in lettuce [47,48,72]. *R. intraradices*-based commercial preparation, especially when supplied together with a legume-derived protein hydrolysate (PH), increased antioxidant activities (CAT and GPX), proline content and SPAD index (correlated to photosynthetic pigments) in lettuce [50]. Proline, being an amino acid accumulated under osmotic stress (under salinity or drought) as an osmolyte, is able to act as an ROS scavenger, protecting and stabilizing membranes and macromolecules and promoting the expression of stress-responsive genes presenting elements responsive to proline [73].

*Funnelliformis mosseae* and *R. irregulare* basil inoculation enhanced phenols, and in particular caffeic and rosmarinic acids and photosynthesis [37], in addition to chlorophylls [38] under non-saline conditions. However, in basil plants under salinity, the same AMF induced the accumulation of polyphenols (i.e., ferulic and chicoric acids and quercetin-rutinoside) [37]. *G. etunicatum* and *G. intraradices*, but in particular *G. fasciculatum*, increased basil essential oils (particularly linalool) [39]; the same *G. fasciculatum* increased also chicoric acid and a caffeic acid derivative in basil [40].

*Glomus intraradices* enhanced lycopene content in Moneymaker tomato fruits; the extracts from these tomatoes did not contain mutagenic compounds [41], and both the hydrophilic and lipophilic fractions of these extracts showed anti-estrogenic power. *R. intraradices* increased polyphenol content in the tomato cultivar Rio Fuego [74]. *F. mosseae* and *R. irregulare* induced increases of carotenoids, antioxidant capacity and volatile compounds but not vitamins in Moneymaker tomato fruits [42]; it also induced increases of lycopene, total ascorbic acid, alanine GABA and branched-chain amino acids in the red cherry tomato Lucariello of the variety “Pomodorino del Piennolo del Vesuvio”, and of the essential amino acids arginine and lysine in the yellow variety [43]. Moreover, the same AMF increased ascorbate content in cherry tomato (var. cerasiforme) [44]. *G. mossae* increased fruit yield and free amino acid content (i.e., glutamine and asparagine) in Micro Tom tomato by upregulating the transcription of genes involved in N and C metabolism [75]. *G. mossae* together with *T. harzianum* increased only lycopene content in tomato fruits [45]. Two commercial preparations, containing 6 and 8 different AMF species, increased the ascorbic acid and total soluble sugars in fruits of AMF-inoculated tomato plants of the variety Admiro F_1_ grown on rockwool, particularly when cultivated with high P [76]. A commercial preparation of *Glomus* sp. increased citric acid in fruits of the tomato variety TC 2000 cultivated on a real industrial tomato farm [77]. *R. irregularis* used on Micro Tom and Brioso tomatoes in industrialized production increased carotenoids, free amino acids and BRIX values up to fourfold [78].

A commercial preparation of *Glomus* sp. increased sweet pepper’s total antioxidant capacity in control plants and yield under water stress [79].

Therefore, AMF-dependent improvement of antioxidant defence in the abovementioned inoculated plants may be correlated with improved photosynthetic capacity, higher yield, better post-harvest storage capacity and premium quality.

Indeed, different plant species respond to AMF inoculation with the activation of different secondary metabolic pathways, and most of the plants undergo an activation of these pathways only in the roots but not in other plant organs, such as edible parts [80]. It can also happen that commercial AMF are not suitable or are not an absolute requirement for all types of soil and agricultural systems. In fact, indigenous AMF populations might yield better results than commercially available inocula [81].

**Table 2:** Vegetable crops’ treatment with AMF-based biostimulants and observed effects.

AMF	Plant Species	Treatments	Observed Effects	Refs
*Funneliformis mosseae* *Claroideoglomus claroideum*	*C. cardunculus* L. cv. romanesco C3 Italy and Violetto Tema	Pots inoculated with crude inoculum of *C. claroideum* 22W3 and/or *F. mosseae* (2W3, IMA1, IN101C)	*C. claroideum* increased total phenols. *C. claroideum* and *F. mosseae* IMA1 increased antioxidant activity.	[64]
*Funneliformis mosseae* *Diversispora versiformis* *Rhizophagus intraradices* *Glomus* sp. (*G. versiforme*, *G. intraradices* and *G. mosseae*)	*Allium cepa* L. Iranian genotypes Red Azar-shahr, White Kashan, Yellow Gholi Ghesse, Pink Horand, and a commercial cultivar Red *Rosita*	Pots inoculated with 50 g AMF crude inoculum mixed into 1 kg of soil, or 50 g sterilized inoculum (control)	AMF increased total phenolics, pyruvic acid, ascorbic acid, flavonol glucosides and antioxidant enzymes, with the highest beneficial effect caused by *D. versiformis*.	[57]
*Funneliformis mosseae* *Rhizoglomus irregulare*	*Lactuca sativa* var. capitata cv. Bolla	Inoculation at transplant with one tablet (225 spores of each AMF, *Trichoderma koningii* TK7 strain at 1 × 10^6^ UFC g^−1^) per pot, under sufficient, moderate or severe deficit irrigation	AMF mix increased P, Mg, Fe, Mn, Zn and phenolic acids independently of water availability. Under well-watered and moderate irrigation, AMF increased plant yield, Ca, Cu and isochlorogenic acid.	[46]
*Funneliformis mosseae* *Rhizoglomus irregulare*	*Ocimum basilicum* L. Gecom	Inoculation at transplant with one tablet (225 spores of each AMF, *T. koningii* TK7 strain at 1 × 10^6^ UFC g^−1^, *Bacillus megaterium* MHBM77 1 × 10⁶ UFC g^−1^ and *B. megaterium* MHBM06 1 × 10⁶ UFC g^−1^) per pot below the basil roots under NaCl 1 or 40 mM NaCl	AMF enhanced the marketable fresh yield, chlorophylls and phenols but decreased nitrate content.	[38]
*Funneliformis mosseae* *Rhizoglomus irregulare*	*Ocimum basilicum* L. Gecom	At transplant with one tablet (225 spores of each AMF, *T. koningii* TK7 strain at 1 × 10^6^ UFC g^−1^, *B. megaterium* MHBM77 1 × 10⁶ UFC g^−1^ and *B. megaterium* MHBM06 1 × 10⁶ UFC g^−1^) per pot below the basil roots	Under low stress, AMF increased photosynthesis, Fe, Mn and caffeic and rosmarinic acids. Under salinity, enhanced Na compartmentalization and P availability, accumulation of polyphenols (i.e., ferulic and chicoric acids and quercetin-rutinoside) but did not change VOC composition.	[37]
*Funneliformis mosseae* *Rhizoglomus irregulare*	*Solanum lycopersicum* L. var. Moneymaker	Pots inoculated with 5% (*v*/*v*) *F. mosseae* and/or *R. irregularis*	AMF increased mineral content (e.g., N, P and Cu), carotenoids, antioxidant capacity and volatile compounds but not vitamins.	[42]
*Funneliformis mosseae* AZ225C *Rhizoglomus irregulare* IMA6	*Lactuca sativa* L. var. crispa	Transplant in peat mixed with crude inoculum (1:5 *v*/*v*)	*R. irregulare* more than *F. mosseae* enhanced concentration of phenolics and antioxidant activity.	[71]
*Funneliformis mosseae* and *Rhizoglomus irregulare* (commercial mix)	*Allium cepa* L. cv. Stuttgarter Riesen	AMF commercial inoculum equally mixed with the quartz sand before seeding or at the start of bulbing	Inoculation before seeding (highest amount) and colonization at late development stages (bulb growth) increased quercetin compounds if plants were additionally supplied with ammonium.	[69]
*Funneliformis mosseae BEG234* and *Rhizoglomus irregulare BEG72* (commercial mix)	*Solanum lycopersicum* L. var. “Pomodorino del Piennolo del Vesuvio” landraces Giagiù (yellow) and Lucariello (red)	Planting holes inoculated with 2 g of commercial microgranular inoculum containing 25 spores g^−1^ of each AM fungus	AMF increased lycopene, total ascorbic acid, alanine GABA and branched-chain amino acids in the red cherry tomato, and Ca, Zn, GABA and the essential amino acids arginine and lysine in the yellow one. In both landraces, AMF improved the antioxidant activity related to the shelf life of tomato fruits.	[43]
*Funneliformis mosseae* BEG 234 and *Rhizoglomus irregulare BEG72* and *Trichoderma koningii* (commercial mix)	*C. cardunculus* subsp. *scolymus* L. *Hayek* cv. cultivars Romolo and Istar	Seed priming with consortium of endophytic fungi containing arbuscular mycorrhizal fungi and *Trichoderma koningii* in a 6:1 ratio	Both microbial-inoculated cultivars showed higher primary and total fresh marketable yields, higher total phenols and higher antioxidant activity.	[66]
*Funneliformis mosseae and Rhizophagus intraradices* (commercial mix) *Rhizophagus intraradices*	*Crocus sativus* L.	10 g of each inoculum were placed under corms before planting	AMF mix increased saffron flower production and yield. *R. intraradices* alone enhanced the content of active metabolites (picrocrocin, crocin II and quercitrin) and antioxidant activity.	[68]
*Funneliformis mosseae* BEG234 and *Rhizoglomus irregulare* BEG72 and (commercial mix) *Streptomyces roseocinereus* MS1B15	*Solanum lycopersicum* L. var. cerasiforme	Pots inoculated with 5 g commercial mix (25 spores g^−1^) in the planting holes and/or by fertigation with 3.33 g of bacterial suspension (5.8 × 10^6^ CFU g^−1^) in 100 mL sterile ddH_2_O	AMF and *S. roseocinereus* solubilized P sources, improved P uptake and content, vegetative growth, total fruit, red fruit number and fruit quality (i.e., colour, shape and Vitamin C).	[44]
*Funneliformis mosseae, Rhizophagus intraradices, Claroideoglomus claroideum, Claroideoglomus etunicatum, G. microaggregatum, Funneliformis geosperum* (2:2:1:1:1:1 mix 1) *Funneliformis mosseae, Rhizophagus inraradices, G. aggregatum, Claroideoglomus etunicatum, G.deserticola, Rhizophagus clarus, G. monosporum* (3:3:3:3:1:1:1:1:1 mix 2)	*Solanum lycopersicum* L. var. Admiro F_1_	Inoculation of plastic covering (before planting) with 10 g of mix 1 (720 propagules g^−1^), and of growing substrate at two-week intervals (14 and 28 DAT) with mix 2 (2.5 g inoculum dm^−3^ water, 160 propagules g^−1^). Each plant received 60 cm^3^ per inoculation. Cultivation with P 15 or 50 mg dm^−3^ and two substrates (i.e., rockwool or coconut coir)	Increase of ascorbic acid and total soluble sugars in fruits of AMF-inoculated plants (particularly with high P) grown on rockwool.	[76]
*Funneliformis *sp., *Claroideoglomus *sp., *Diversispora *sp., *Glomus *sp. and *Rhizophagus *sp. (commercial mix) *Glomus intraradices*, *G. microageregatum* BEG and *G. Claroideum* BEG 210 (commercial mix) *Funneliformis mosseae* (Fm)	*Cucumis sativus* L. cv. Zhongnong No. 106	Pots inoculated with 10 g of crude inoculum of commercial mixes or *F. mossaeae* containing about 2200 infective propagules g^−1^ from infected cultures	AMF increased plant growth, photosynthetic activity and macro- and micronutrient use efficiency.	[51]
*Glomus etunicatum* *Glomus fasciculatum* *Glomus intraradices*	*Ocimum basilicum* L.	Holes inoculated with 5 g per seed of AMF before planting	Increase of root dry weight, leaf area, plant height and number of lateral branches, minerals (N, P, K, Ca, Fe, Cu and Mn). *G. fasciculatum* increased yield and essential oils (particularly linalool) more than other AM fungi.	[39]
*Glomus etunicatum, G. microaggregatum, G. intraradices, G. claroideum, G. mosseae* and *G. geosporum* (commercial mix)	*Capsicum annuum* L. cv. SLAVY F_1_	Inoculation of seedling substrate with commercial mix at 10% concentration with two levels of irrigation (optimum and stress)	AMF increased yield under water stress and increased total antioxidant capacity in control plants.	[79]
*Glomus etunicatum*, *G. microaggregatum*, *G. intraradices*, *G. claroideum*, *G. mosseae* and *G. geosporum* (commercial mix) *G. intraradices* BEG140	*Allium cepa* L. cv. Alice	Planting holes inoculated with 120 g of crude inoculum of commercial AMF mix or *G. intraradices* BEG140, originated from infected maize plants in presence of bark chips preinoculated with saprotrophic fungi	AMF mix increased growth (100%) more than *G. intraradices* alone (50%). *G. intraradices* alone increased Mg and K in bulb tissue, and S in presence of saprotrophic fungi.	[53]
*Glomus fasciculatum*	*Ocimum basilicum* L. cv. Cinnamon, Siam Queen, Sweet Dani and Red Rubin	Pots inoculated with 50 g of crude inoculum (15 propagules g^−1^ soil substrate) before sowing seeds with 64 or 128 mg·L^−1^ P	AMF inoculation enhanced N, K, S, B, Fe and Zn uptake, and the content of phenolics (chicoric acid and a caffeic acid derivative). AMF and high P increased biomass and P content.	[40]
*Glomus fasciculatum* Commercial mix of *Glomus intraradices* and *Glomus mosseae*	*Lactuca sativa* L. var. capitata or longifolia	Pots inoculated with 2 g each of *G. fasciculatum* infected alfalfa soil (mycorrhizal roots and soil containing spores and extraradical mycelium) or commercial mix.	Increase in minerals, chlorophylls, carotenoids, starch and soluble sugars, proteins, ascorbate and tocopherol, phenolics and growth.	[47,48,82]
*Glomus intraradices*	*Solanum lycopersicum* L. var. Moneymaker	Pots inoculated with 30 g of crude inoculum from infected pot cultures and 50 mL of a filtrate of mycorrhizal inoculum (50 μm pore ø) to all treatments, included controls, to ensure common microflora	AMF increased growth, mineral nutrients of plants and lycopene in fruits. The extracts from these tomatoes did not contain mutagenic compounds; both the hydrophilic and lipophilic fractions of these extracts showed anti-estrogenic power.	[41]
*Glomus intraradices* and *Glomus mosseae* (commercial mix)	*Lactuca sativa* L. var. capitata	Pots inoculated under 100%, 75% or 50% water field capacity (FC)	Normal plant growth under 75% FC, increase of carotenoids, anthocyanins and to a lesser extent chlorophylls and phenolics	[72]
*Glomus intraradices* *Glomus mosseae*	*C. cardunculus* L. var. scolymus	Offshoot inoculated with 50 g of crude inoculum originated from infected pot cultures of each or both AMF species	Each species, but even more so both species together, were able to increase total phenolic content and antioxidant activity.	[65]
*Glomus intraradices* with low concentrations of *Trichoderma* *harzianum* and *Bacillus subtilis* (commercial mix)	*Allium sativum* L. cultivar Maysky *Allium cepa* L. cultivar Kaba	AMF double inoculation before planting and at beginning of bulb formation and/or foliar supply of sodium selenate	AMF + Se increased (i) yield, monosaccharides, P, K and Se in both garlic and onion bulbs; (ii) ascorbic acid, flavonoids, Mg and microelements (B, Cu, Fe, Mn, Si and Zn) in onion; (iii) flavonoids in garlic.	[54,55]
*Glomus mosseae*	*Coriander sativum* L.	Pots inoculated with 100 g of *G. mosseae* crude inoculum from infected Sudan grass with 0/100 mg kg^−1^ KH_2_PO_4_	AMF increased growth, P and N in shoot and root tissues, total soluble proteins in root tissues, fruit yield and essential oil contents. Addition of P reduced AMF colonization and its beneficial effects.	[52]
*Glomus mosseae*	*Solanum lycopersicum* L.	Inoculation with 10 g *G. mosseae* commercial granulate kg^−1^ peat before sowing and/or *Trichoderma harzianum* applied at sowing or two weeks later as wettable powder to reach a population of 1.8 × 10^7^ conidia g^−1^ peat	AMF increased the percentage of extra-large fruit, while *T. harzianum* inoculated two weeks after sowing decreased Ca and Mg in tomato fruit. AMF and *T. harzianum* increased total and marketable yield and lycopene of tomato fruits, but not other antioxidant metabolites or antioxidant activity.	[45]
*Glomus mosseae*	*Solanum lycopersicum* cv. Micro Tom	Inoculation by mixing AMF commercial inoculum and sand (30:70, *v*/*v*)	Increase of fruit yield and free amino acid content (i.e., glutamine and asparagine). Upregulation of transcription of genes involved in N and C metabolism.	[75]
*Rhizophagus irregularis*	*Allium cepa* cv. Karmen, Kuba, Sochaczewska and Wolska	Inoculation of upper layer of substrate (15 g of commercial inoculum per pot)	AMF improved onion photosynthesis, growth and yield, and increased vitamin B1 and organic acids.	[70]
*Rhizophagus intraradices*	*Solanum lycopersicum* L. cv. Rio Fuego	Priming with (3 g L^−1^ (~ 100 spores g^−1^) AMF and/or 50 mL of 0.8% seaweed extract (SE; *Padina gymnospora*) and/or watering with nutritive solution. Control groups treated only with water.	AMF increased polyphenol content. SE favoured protein content. AMF + SE accelerated flowering and AMF colonization and increased root and shoot growth, protein and carbohydrate content.	[74]
*Rhizophagus intraradices*	*Lactuca sativa* cv. Meraviglia d’Inverno	Inoculation at transplant under roots with one tablet (containing 200 spores of *R. intraradices* BEG72 and 4.5 × 10^7^ CFU of *T. atroviride* MUCL45632) and/or 2.5 mL L^−1^ legume-derived PH foliar spray (4 times at weekly intervals from 6 DAT) and standard, saline (25 mM NaCl) or alkaline (10 mM NaHCO_3_ + 0.5 g L^−1^ CaCO_3_) solution	AMF tablet, especially with PH, improved fresh marketable yield, dry weight, SPAD index, antioxidant activities (CAT and GPX), proline, P, K and Fe via an increase of total root length and surface.	[50]
*Rhizophagus intraradices*	*Lactuca sativa* cv. Valeska	Peat substrate inoculated within AMF (4.25 g L^−1^, 720 propagules g^−1^), medium with P 70 or 140 mg dm^−3^ and Se in the substrate 0, 6 or 12 mg dm^−3^	AMF in plants under low P concentration improved yield but did not affect Se or sugar accumulation.	[49]
*Rhizophagus intraradices BEG72, Funneliformis mosseae* and *Trichoderma* *atroviride* (commercial mix)	*C. cardunculus* subsp. *scolymus* L. *Hayek* cv. cultivars Romolo and Istar	Seed coating at 6:1 ratio with commercial mix (300 spores g^−1^ *R. intraradices,* 200 spores g^−1^ *Funneli-* *formis mosseae*, and 3 × 10^8^ CFU *Trichoderma* *atroviride*) planted in September or October	AMF mix increased the content of 3-O-caffeoylquinic acid, 5-O-caffeoylquinic acid and apigenin 7-O-glucuronide in primary heads as well 1,5-di-O-caffeoylquinic acid in secondary heads especially in Romolo cv.	[67]
*Rhizophagus intraradices*, *G. aggregatum*, *G. viscosum*, *Claroideoglomus etunicatum* and *Claroideoglomus claroideum* (commercial mix)	*Solanum lycopersicum* var. TC 2000 cultivated in real industrial tomato farm	Alveolar boxes inoculated with 20 mL of commercial mix inoculum (85,000 infective propagules/l or 10 mL of two *Pseudomonas* bacterial suspension (10^8^ CFU mL^−1^))	AMF mix increased citric acid concentration, while bacteria positively modulated the sugar production and the sweetness of the tomatoes. Both treatments allowed the reduction of chemical inputs and positively influenced tomato quality.	[77]
*Rhizophagus irregularis* strain K8/QS69	*Solanum lycopersicum* cv. Micro Tom, Brioso	Inoculation of one-week-old seedlings or cuttings with AMF after enrichment by previous co-cultivation with leek, with 2.7, 6.7 and 10.7 mM phosphate	AMF enhanced the nutritional value of tomatoes in industrialized production by increasing BRIX values, carotenoids and free amino acids (up to fourfold).	[78]
*Rhizophagus irregularis* WFVAM10	*Solanum lycopersicum* L. cv. 76R, *Lactuca sativa* L., *Daucus carota* L., *Cucumis sativus* L., *Allium ampeloprasum* L. var. *porrum* and other legumes and cereals	Soil mixed with AMF crude inoculum (9:1 *w*/*w*) after enrichment by previous co-cultivation with clover	AMF increased the content of P, Cu, Zn and S in shoots and edible parts (where present) of plants, and in particular in leek, whose biomass was also enhanced. The mineral content (N, P, S and Cu) of carrot was also highly increased. Plant ionome was more affected by plant species than by inoculation with AMF.	[36]

## 5. Plant Growth-Promoting Bacteria (PGPB)

PGPB include both bacteria which live freely in the soil and rhizobacteria which colonize the rhizosphere. The beneficial effects of these microorganisms depend mainly on their capacity to solubilize inorganic nutrients and synthetise plant growth regulators [83]. Many PGPB species have been actively studied to investigate their potential role as biostimulants on the yield and quality of vegetable crops, and some of them have already been commercialized, but only *Rhizobium, Azospirillum* and *Azotobacter* genera have been approved by Regulation (EU) 2019/1009 [26].

Indeed, the majority of studies on *Rhizobium* sp. are focused on their symbiotic association with legumes and their ability to reduce atmospheric nitrogen and provide organic nitrogen as amides to plants [83,84]. However, these microorganisms can positively interfere with plants’ hormonal balance by producing phytohormones like indole acetic acid (IAA) or the enzyme ACC deaminase, which is involved in the metabolism of 1-aminocyclopropane-1-carboyclic acid (ACC), a precursor of ethylene; they can also mobilize soil-insoluble nutrients (e.g., phosphates) supplying plants with soluble ones (e.g., phosphorus) [83]. Moreover, rhizobia are able to synthetise Fe^3+^-chelating molecules, called siderophores, that inhibit the growth of phytopathogens. These molecules can be citric acid or β-hydroxy aspartic acid, having carboxyl and hydroxyl groups, which bind available Fe^3+^ with high affinity to form complexes which are internalized by the cells with the help of cognate membrane proteins [85,86]. Plant growth-promoting rhizobacteria (PGPR) able to produce active siderophores can spoil iron from other strains, thus suppressing other soil-borne plant pathogens [86]. In addition, PGPR can also elicit a defence-stimulating effect both in roots and leaves, called induced systemic resistance (ISR), against pathogens and insects [87]. In particular, rhizobia by releasing siderophores induce Fe deficiency in the host plants and solicit them to release coumarins, which are phenolic compounds that favour Fe acquisition by roots while functioning as antimicrobial against soil-borne pathogens but not the rhizobia [87]. However, mutants unable to synthetise siderophores, by interfering with plant ethylene biosynthesis and signalling pathways, can elicit the Fe deficiency response and ISR in plants. In fact, ethylene, through the activation of the transcription factors FIT, bHLH38 and bHLH39, can upregulate the expression of several genes associated with the Fe deficiency and the secretion of phenolic compounds, like coumarins. In addition to ethylene, auxin and nitric oxide (NO) can also upregulate Fe-related genes and ISR [88]. Given all these beneficial effects, the application of PGPR as biostimulants has increased in recent years, even if their use for stimulating plant yield and quality in vegetable crops is quite recent (Table 3) except for a few isolated cases, including that of Abdelgani, Elsheikh and Mukhtar [30]. Camelo et al. [89] reported that rhizobacteria interact with the roots of non-legume plants through chemotaxis mechanisms, as it happens with legume plants; in fact, they are attracted by substances extruded by the root which direct their movement towards roots, initiating a beneficial symbiosis. Among the few new articles dealing with PGPR and vegetable crops (Table 3), that of García-Fraile et al. [90] showed that the inoculation of seedlings of cherry tomato and Verde Italiano sweet pepper with *Rhizobium legiminosarum* strain TPV08 and *Rhizobium* sp. strain PETP01 positively affected plant growth. In addition, while the beneficial effects in pepper were more linked to fruit production, whose fresh weight was significantly increased, the ones in tomato were more related to their quality, with a significant increase in mineral content (N, P, K or Mg). The inoculation of seedlings with *Rhizobium laguerreae* strain HUTR05 increased N and P content, phenolic acids (e.g., dicaffeoyl quinic and cichoric acids) and increased quercetin 3-O-glucoside flavonoid content in romaine lettuce [91]. The use of *Rhizobium laguerreae* strain PEPV40 in spinach increased leaf number, size and weight, as well as chlorophyll and nitrogen contents [84]. The same bacterial strain together with *Bacillus halotolerans* SCCPVE07 promoted plant development even under salinity and enhanced the contents of K, Fe, Mg, N, phenolic acids (cichoric acid and caffeoyl-tartaric acid) and flavonoids (kaempferol 3-O-glucuronide) in endive [92].

*Azospirillum* and *Azotobacter* are free-living nitrogen-fixing bacteria that have been frequently used in inoculant products. In particular, *Azospirillum*, which is among the most-studied genera of PGPB in the world, has been used (particularly *A. brasiliense*) as a base for more than 100 biostimulant products in South America alone [93], most of which, but not all, are registered for use in wheat and maize [94]. The beneficial effects exerted by the bacteria of this genus on plant growth depend not only on nitrogen fixation and phosphate solubilization, but also on the synthesis of phytohormones, plant regulators (among which are a variety of molecules with low molecular weight) and enzymes that enhance membrane activity and proliferation of the root system, thus improving the uptake of water and ions and reducing the effects of abiotic stress and pathogen infections (ISR response). This has led to the multiple mechanisms hypothesis, which posits that it is not a single mechanism among those listed above that promotes plant growth, but rather a combination of a few or many of them exerts this positive action [94]. However, other rhizobia, currently not considered belonging to the genus Rhizobium, are able to act as microbial biostimulants in plants. In fact, Xu et al. [95] recently found that co-inoculation of *Azorhizobium caulinodans* and *Piriformospora indica* improved the growth and fruit quality of tomato under salt stress. Chanratana et al. [96] showed that chitosan-immobilized aggregated *Methylobacterium oryzae* CBMB20 improved the physiological state of tomato plants under salinity. Native bacteria isolated from the roots and rhizosphere of *Solanum lycopersicum* L., including a strain similar to *Ochrobactrum anthropi*, increased the growth of tomato seedlings even under reduced fertilization [97]. *Sinorhizobium meliloti* of the wild type and genetically modified derivative strains showed a growth-promoting effect on lettuce in specific interaction with *G. mosseae* or *G. intraradices* [98]. *Sinorhizobium*, formerly (40 years ago) *Rhizobium, meliloti,* is now classified as a member of the Ensifer genus [99]. In addition, there are patents related to rhizobia inoculants and vegetable yield, such as US11147276 (including *Methylobacterium*) [100], that were not retrievable from the used databases because they do not include documents from specialized patent applications databases, such as Derwent Innovation (https://clarivate.com/products/ip-intelligence/patent-intelligence-software/derwent-innovation accessed on 24 August 2022) or Espacenet (https://worldwide.espacenet.com accessed on 24 August 2022). In addition, a few recent articles report the use of *Azospirillum* and *Azotobacter* for improving the quality and yield of vegetable crops even under stress conditions (Table 3). In fact, Fasciglione et al. [101] reported that the inoculation of lettuce seeds with *Azospirillum brasilense* Sp245 increased plant survival of transplantation under salinity (40 mM NaCl) and enhanced the fresh and dry leaf weight, leaf area, chlorophyll and ascorbic acid content. The fresh yield of two lettuce cultivars (Santoro and Quintus) was also improved by an *Azospirillum*- and *Azotobacter*-based bacterial–algal mix, even if carotenoids and antioxidant activity were enhanced only in only in the cultivar Quintus (romaine lettuce) [102,103]. Kolega et al. [104] inoculated two cultivars of basil plants with *Azospirillum brasilense* Cd (DSM-1843), determining increases in root growth, unsaturated fatty acids, flavonoids, alkaloids and several terpene derivatives, particularly in the Red Rubin cultivar. However, fresh yield was enhanced by additional N or S nutrition but not by the nitrogen-fixing bacteria [104]. The inoculation of basil with a commercial preparation of *Azospirillum brasilense* and *Azotobacter chroococcum* increased the plant’s fresh and dry yield independently of intercropping with maize. Moreover, the both the cultivation with 100% N rate and that with 50% N rate plus the inoculum were able to enhance the content of methyl chavicol, an important component of basil essential oil and nutraceutical quality [105]. *Azospirillum lipoferum* DO12 inoculated in the rhizosphere of the tomato cultivar Menhir F_1_ was able to improve the premium quality, and most likely the shelf life, of tomatoes by enhancing their contents of lycopene, Vitamin C and total polyphenols [106]. An *Azospirillum* sp. and *Azotobacter* sp. commercial preparation, inoculated 15 days after transplanting, increased cherry tomato growth and yield, fruit dry matter content, acidity and total soluble contents even under salinity [107]. The inoculation of Pimiento pepper with *Azospirillum* sp. and *G. intraradices* increased pepper fruit Vitamin C, total soluble solids and acidity index; moreover, it improved N and P uptake when a nutrient solution was supplied at half of the normal rate of N and P [108]. The seed-priming of fennel cv. Isfahan with *Azospirillum* strains *lipoferum*, *brasilense*, *irakense* and strain 21 increased seed weight uniformly, as well as essential oil yield, in particular α-pinene and limonene. However, β-pinene but not limonene increased only when the inoculum was done with *Azospirillum* strain 21 [109].

**Table 3:** Vegetable crops’ treatment with PGPB-based biostimulants and observed effects.

PGPB	Plant Species	Treatments	Observed Effects	Refs
*Azospirillum brasilense* Sp245	*Lactuca sativa* L. cv. Elisa	Seeds inoculated with 10^9^ CFU per seed or phosphate buffer, and plants grown under salinity (0–40 mM NaCl)	Increase of plant survival of transplantation at 40 mM NaCl and enhancement of fresh and dry leaf weight, leaf area, chlorophyll and ascorbic acid content	[101]
*Azospirillum brasilense* Cd (DSM-1843)	*Ocimum basilicum* L. cv. Genovese and Red Rubin	Plants inoculated twice with bacteria 10^6^ CFU mL^−1^ in the nutrient solution and/or with additional 20 mM NO_3_^–^ or 8 mM SO4^2−^	Additional nutrients but not *A. brasilense* enhanced fresh biomass. Inoculation increased root growth, unsaturated fatty acids, flavonoids, alkaloids and several terpene derivatives, particularly in Red Rubin.	[104]
*Azospirillum brasilense* and *Azotobacter chroococcum* (commercial mix)	*Ocimum basilicum* L.	Inoculation of soil or seed soaking and application to soil (2 l ha^−1^ with 10^8^ CFU mL^−1^), bacteria + 50% N or 100% N with and without intercropping with maize	Bacteria application increased fresh and dry yield independently of cropping system. 100% N and bacteria + 50% N were both effective in increasing essential oil (methyl chavicol).	[105]
*Azotobacter *sp., *Azospirillum *sp., *Bacillus licheniformis, B. megatheriumstrain Herbaspirillum *sp. and *Chlorella vulgaris* (commercial mix)	*Lactuca sativa* var. crispa L. cv. Santoro and var. longifolia Lam. cv. Quintus	Application of 0.4 l of bacterial and algal mix per plant every 14 days, for a total of four treatments	Bacterial–algal mix increased the weight of both lettuce varieties but increased total carotenoid and antioxidant activity only in the cv. Quintus (romaine lettuce).	[102,103]
*Azospirillum lipoferum* DO12 and *Brevibacillus parabrevis* B50	*Lycoperson esculentum Mill.* cv. *Menhir F_1_*	Rhizosphere inoculation with 25 g m^−2^ *A. lipoferum* (2 × 10^8^ CFU g^−1^), or *B. parabrevis* (3 × 10^9^ CFU g^−1^)	Both bacteria increased tomato marketable yield. *A. lipoferum* enhanced lycopene, Vitamin C and total polyphenols; *B. parabrevis* increased mainly polyphenols.	[106]
*Azospirillum* sp. and *Azotobacter* sp. (commercial mix)	*Solanum lycopersicum* L. var. cerasiforme	Plastic bag inoculation with 1.4 l of solution prepared with 1 mL L^−1^ of commercial mix (1.3 × 10^7^ CFU mL^−1^ of *Azospirillum* and 5.9 × 10^7^ CFU mL^−1^ of *Azotobacter*) with different levels of NaCl (0, 50, 100, 150 mM)	Bacterial mix improved plant growth and yield, fruit dry matter content, pH 4.52, and TSS even under salinity.	[107]
*Azospirillum* sp. *G. intraradices*	*Capsicum annuum* L. (Chile Morrón, Pimiento)	Inoculation with *Azospirillum *sp. 10^4^ and 10^6^ CFU mL^−1^ in the nutrient solution at transplant and twice every 30 days, and 25 or 50 spores of *G. intraradices* at transplant with 50%/100% N and P	Higher concentration of spores and bacteria increased Vitamin C, carotenoids, total soluble solids and acidity; moreover, they improved N and P uptake at reduced N rate.	[108]
*Azospirillum* strains (*lipoferum*, *brasilense*, *irakense* and strain 21)	*Foeniculum vulgare* cv. Isfahan	Seed-priming with *Azospirillum* solution (4 mL g^−1^) × 12 h or microelements	Priming increased seed weight uniformly, essential oil yield, in particular α-pinene and limonene, and in strain 21 also β-pinene but not limonene.	[109]
*Rhizobium laguerreae* strain HUTR05	*Lactuca sativa* L. var. romaine	Seedling inoculation with 150 µL of bacterial suspension with 10^8^ CFU mL^−1^	It increased N and P content, phenolic acids (e.g., dicaffeoyl quinic and cichoric acids) and quercetin 3-O-glucoside flavonoid.	[91]
*Rhizobium laguerreae* strain PEPV40	*Spinacia oleracea* L.	Inoculation of each seedling at the intersection between roots cotyledons with 250 μL of suspension (108 CFU mL^−1^)	Increase of spinach leaf number, size and weight, as well as chlorophyll and nitrogen contents.	[84]
*Rhizobium laguerreae* strain PEPV40 and *Bacillus halotolerans* SCCPVE07	*Cichorium endivia* L.	Plants inoculated with 2 mL of bacterial suspension (10^8^ CFU mL^−1^) and irrigated with water containing 0 or 100 mM NaCl	Bacteria promoted plant development even under salinity. They increased K, Fe, Mg, N, phenolic acids (cichoric acid and caffeoyl-tartaric acid) and flavonoids (kaempferol 3-O-glucuronide).	[110]
*Rhizobium legiminosarum* strain TPV08 *Rhizobium* sp. strain PETP01	*Solanum lycopersicum* L. var. Cherry *Capsicum annuum* L. var. Verde Italiano	Seedlings inoculated with 10^8^ CFU of each strain	TPV08 and PETP01 promoted growth of both tomato and pepper, but particularly pepper fresh weight production and tomato quality (higher N, P, K or Mg).	[90]
*Rhizobium etli* CE-3, *R. leguminosarum SCR* *R. leguminosarum Semia—4088.*	*Solanum lycopersicum L.*	Seed priming with 4 mL of each inoculum (10^8^ CFU mL^−1^) kg^−1^ seeds + inoculation at 30 DAS with 10% of the covering of the root balls in each treatment	Rhizobia (particularly etli CE-3 and Rl SCR) improved tomato yield, probably by a more efficient acquisition of N, P and K. There were no monetary losses despite the different effects.	[111]

## 6. Conclusions

**Figure 2:**
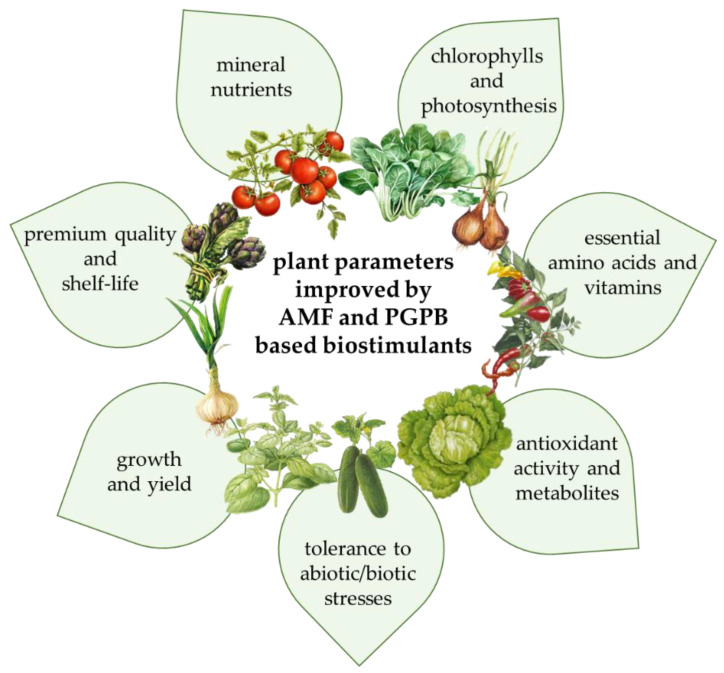
Main beneficial effects exerted by AMF and PGPB on vegetable crops in relation to yield and nutritional quality.

The inoculation with beneficial microorganisms (AMF and/or PGPB) of vegetable crops grown under both open-field and greenhouse conditions allowed the production of high-quality foods, enhancing the concentration of functional compounds such as secondary metabolites as well as minerals and micronutrients that are well-recognized for their health-promoting properties. As shown above, AMF and PGPB can enhance plant growth and functional quality by increasing the uptake of mineral nutrients, the production of metabolites (among which are essential amino acids, carotenoids and polyphenols,) and the activity of antioxidant enzymes [112]. These effects, even differently modulated by diverse AMF isolates and bacterial strains and also depending on the vegetable species or cultivar [113], cause an increase of photosynthetic performance and yield, exerting a positive effect on yield and premium quality and possibly also on shelf life (Figure 2). The semi-systematic review indicates that the effects of microbial biostimulant application on the yield and functional quality of vegetables depend not only on the microbial strains, but also on the application management as well as on the environment conditions. Therefore, future research should be focused on: (1) elucidating the microbial biostimulant strain × species/cultivar × environment interaction in order to select the best combination(s); (2) identifying new PGPB and AMF strains that interact synergistically to boost the yield and functional quality of the selected vegetables; and (3) understanding the physiological and molecular mode of the actions behind the enhancement of nutritional and functional quality parameters in vegetable products induced by microbial biostimulant application.

## Figures and Tables

**Table 1:** Article screening criteria and key words.

Criterion	Keywords
Microbe or microbial biostimulant	‘microbial biostimulant’ (and/or ‘Azotobacter’, ‘Mycorrhiza’/‘Mycorrhizal’, ‘Rhizobium’, ‘Azospirillum’)
Parameter	‘food quality’ and/or ‘nutritional quality’ and ‘yield’

## Data Availability

The data are contained within the article and in the Supplemental File S1.

## Supplementary Materials

The following supporting information can be downloaded at: https://www.mdpi.com/article/10.3390/foods11172656/s1, Supplemental File S1: List of articles initially screened for inclusion.

## References

[1] Klein Goldewijk K., Beusen A., van Drecht G., de Vos M. (2011). The HYDE 3.1 spatially explicit database of human-induced global land-use change over the past 12,000 years. Glob. Ecol. Biogeogr..

[2] Worldmeter World Population Clock. https://www.worldometers.info/world-population/.

[3] AsiaNews (2017). About a Billion People Are Invisible, One Third of Them Children. AsiaNews Agencies.

[4] Simson K. (2022). The Value of Early Action on Energy Efficiency. Speech by Commissioner Simson at the IEA’s 7th Annual Global Conference on Energy Efficiency Opening Plenary. SPEECH/22/3546. https://ec.europa.eu/commission/presscorner/detail/en/SPEECH_22_3546.

[5] Carillo P., Colla G., El-Nakhel C., Bonini P., D’Amelia L., Dell’Aversana E., Pannico A., Giordano M., Sifola M.I., Kyriacou M.C. (2019). Biostimulant Application with a Tropical Plant Extract Enhances Corchorus olitorius Adaptation to Sub-Optimal Nutrient Regimens by Improving Physiological Parameters. Agronomy.

[6] Carillo P., Colla G., Fusco G.M., Dell’Aversana E., El-Nakhel C., Giordano M., Pannico A., Cozzolino E., Mori M., Reynaud H. (2019). Morphological and Physiological Responses Induced by Protein Hydrolysate-Based Biostimulant and Nitrogen Rates in Greenhouse Spinach. Agronomy.

[7] Colla G., Rouphael Y. (2015). Biostimulants in horticulture. Sci. Hortic..

[8] du Jardin P. (2015). Plant biostimulants: Definition, concept, main categories and regulation. Sci. Hortic..

[9] Sangiorgio D., Cellini A., Donati I., Pastore C., Onofrietti C., Spinelli F. (2020). Facing climate change: Application of microbial biostimulants to mitigate stress in horticultural crops. Agronomy.

[10] Rouphael Y., Colla G. (2020). Toward a sustainable agriculture through plant biostimulants: From experimental data to practical applications. Agronomy.

[11] Emmanuel O.C., Babalola O.O. (2020). Productivity and quality of horticultural crops through co-inoculation of arbuscular mycorrhizal fungi and plant growth promoting bacteria. Microbiol. Res..

[12] Scagliola M., Valentinuzzi F., Mimmo T., Cesco S., Crecchio C., Pii Y. (2021). Bioinoculants as Promising Complement of Chemical Fertilizers for a More Sustainable Agricultural Practice. Front. Sustain. Food Syst..

[13] Lucini L., Rouphael Y., Cardarelli M., Canaguier R., Kumar P., Colla G. (2015). The effect of a plant-derived biostimulant on metabolic profiling and crop performance of lettuce grown under saline conditions. Sci. Hortic..

[14] Ertani A., Cavani L., Pizzeghello D., Brandellero E., Altissimo A., Ciavatta C., Nardi S. (2009). Biostimulant activity of two protein hydrolyzates in the growth and nitrogen metabolism of maize seedlings. J. Plant Nutr. Soil Sci..

[15] Rouphael Y., Colla G. (2018). Synergistic Biostimulatory Action: Designing the Next Generation of Plant Biostimulants for Sustainable Agriculture. Front. Plant Sci..

[16] Colla G., Hoagland L., Ruzzi M., Cardarelli M., Bonini P., Canaguier R., Rouphael Y. (2017). Biostimulant Action of Protein Hydrolysates: Unraveling Their Effects on Plant Physiology and Microbiome. Front. Plant Sci..

[17] Turner T.R., James E.K., Poole P.S. (2013). The plant microbiome. Genome Biol..

[18] Singh S.K., Wu X., Shao C., Zhang H. (2022). Microbial enhancement of plant nutrient acquisition. Stress Biol..

[19] Dell’Aversana E., D’Amelia L., De Pascale S., Carillo P., Hasanuzzaman M. (2020). Use of Biostimulants to Improve Salinity Tolerance in Agronomic Crops. Agronomic Crops: Volume 3: Stress Responses and Tolerance.

[20] Nishad R., Ahmed T., Rahman V.J., Kareem A. (2020). Modulation of Plant Defense System in Response to Microbial Interactions. Front. Microbiol..

[21] Owen D., Williams A.P., Griffith G.W., Withers P.J.A. (2015). Use of commercial bio-inoculants to increase agricultural production through improved phosphrous acquisition. Appl. Soil Ecol..

[22] Etesami H., Jeong B.R., Glick B.R. (2021). Contribution of Arbuscular Mycorrhizal Fungi, Phosphate-Solubilizing Bacteria, and Silicon to P Uptake by Plant. Front. Plant Sci..

[23] Begum N., Qin C., Ahanger M.A., Raza S., Khan M.I., Ashraf M., Ahmed N., Zhang L. (2019). Role of Arbuscular Mycorrhizal Fungi in Plant Growth Regulation: Implications in Abiotic Stress Tolerance. Front. Plant Sci..

[24] Zeilinger S., Gupta V.K., Dahms T.E., Silva R.N., Singh H.B., Upadhyay R.S., Gomes E.V., Tsui C.K., Nayak S.C. (2016). Friends or foes? Emerging insights from fungal interactions with plants. FEMS Microbiol. Rev..

[25] Zaidi A., Ahmad E., Khan M.S., Saif S., Rizvi A. (2015). Role of plant growth promoting rhizobacteria in sustainable production of vegetables: Current perspective. Sci. Hortic..

[26] EC (2019). REGULATION (EU) 2019/1009 of the European Parliament and of the Council of 5 June 2019 Laying down Rules on the Making Available on the Market of EU Fertilising Products and Amending Regulations (EC) No 1069/2009 and (EC) No 1107/2009 and Repealing Regulation (EC) No 2003/2003. https://eur-lex.europa.eu/legal-content/EN/TXT/?uri=celex%3A32019R1009.

[27] Barros-Rodriguez A., Rangseekaew P., Lasudee K., Pathom-aree W., Manzanera M. (2020). Regulatory risks associated with bacteria as biostimulants and biofertilizers in the frame of the European Regulation (EU) 2019/1009. Sci. Total Environ..

[28] Hendriksen N.B. (2022). Microbial biostimulants—The need for clarification in EU regulation. Trends Microbiol..

[29] Moher D., Liberati A., Tetzlaff J., Altman D.G. (2009). Preferred reporting items for systematic reviews and meta-analyses: The PRISMA statement. BMJ.

[30] Abdelgani M.E., Elsheikh E.A.E., Mukhtar N.O. (1999). The effect of Rhizobium inoculation and chemical fertilization on seed quality of fenugreek. Food Chem..

[31] Kapoor R., Giri B., Mukerji K.G. (2002). Mycorrhization of coriander (*Coriandrum sativum* L.) to enhance the concentration and quality of essential oil. J. Sci. Food Agric..

[32] Rouphael Y., Franken P., Schneider C., Schwarz D., Giovannetti M., Agnolucci M., Pascale S.D., Bonini P., Colla G. (2015). Arbuscular mycorrhizal fungi act as biostimulants in horticultural crops. Sci. Hortic..

[33] Chen M., Arato M., Borghi L., Nouri E., Reinhardt D. (2018). Beneficial Services of Arbuscular Mycorrhizal Fungi—From Ecology to Application. Front. Plant Sci..

[34] Rouphael Y., Kyriacou M.C., Petropoulos S.A., De Pascale S., Colla G. (2018). Improving vegetable quality in controlled environments. Sci. Hortic..

[35] Baum C., El-Tohamy W., Gruda N. (2015). Increasing the productivity and product quality of vegetable crops using arbuscular mycorrhizal fungi: A review. Sci. Hortic..

[36] Tran B.T.T., Watts-Williams S.J., Cavagnaro T.R. (2019). Impact of an arbuscular mycorrhizal fungus on the growth and nutrition of fifteen crop and pasture plant species. Funct. Plant Biol..

[37] Saia S., Corrado G., Vitaglione P., Colla G., Bonini P., Giordano M., Di Stasio E., Raimondi G., Sacchi R., Rouphael Y. (2021). An Endophytic Fungi-Based Biostimulant Modulates Volatile and Non-Volatile Secondary Metabolites and Yield of Greenhouse Basil (*Ocimum basilicum* L.) through Variable Mechanisms Dependent on Salinity Stress Level. Pathogens.

[38] Rouphael Y., Colla G., Giordano M., Raimondi G., Pannico A., Di Stasio E., Cardarelli M., Bonini P., de Pascale S. (2020). Endophytic fungi induce salt stress tolerance in greenhouse-grown basil. Acta Hortic..

[39] Rasouli-Sadaghiani M., Hassani A., Barin M., Rezaee Danesh Y., Sefidkon F. (2010). Effects of arbuscular mycorrhizal (AM) fungi on growth, essential oil production and nutrients uptake in basil. J. Med. Plants Res..

[40] Scagel C.F., Lee J. (2012). Phenolic Composition of Basil Plants Is Differentially Altered by Plant Nutrient Status and Inoculation with Mycorrhizal Fungi. HortScience Horts.

[41] Giovannetti M., Avio L., Barale R., Ceccarelli N., Cristofani R., Iezzi A., Mignolli F., Picciarelli P., Pinto B., Reali D. (2012). Nutraceutical value and safety of tomato fruits produced by mycorrhizal plants. Br. J. Nutr..

[42] Hart M., Ehret D.L., Krumbein A., Leung C., Murch S., Turi C., Franken P. (2015). Inoculation with arbuscular mycorrhizal fungi improves the nutritional value of tomatoes. Mycorrhiza.

[43] Carillo P., Kyratzis A., Kyriacou M.C., Dell’Aversana E., Fusco G.M., Corrado G., Rouphael Y. (2020). Biostimulatory Action of Arbuscular Mycorrhizal Fungi Enhances Productivity, Functional and Sensory Quality in ‘Piennolo del Vesuvio’ Cherry Tomato Landraces. Agronomy.

[44] Chouyia F.E., Fiorentino N., Rouphael Y., Ventorino V., Fechtali T., Visconti D., Cozzolino E., Idbella M., Giordano M., Fagnano M. (2022). Assessing the effect of P-solubilizing bacteria and mycorrhizal fungi on tomato yield and quality under different crop rotations. Sci. Hortic..

[45] Nzanza B., Marais D., Soundy P. (2012). Yield and nutrient content of tomato (*Solanum lycopersicum* L.) as influenced by Trichoderma harzianum and Glomus mosseae inoculation. Sci. Hortic..

[46] Saia S., Colla G., Raimondi G., Di Stasio E., Cardarelli M., Bonini P., Vitaglione P., De Pascale S., Rouphael Y. (2019). An endophytic fungi-based biostimulant modulated lettuce yield, physiological and functional quality responses to both moderate and severe water limitation. Sci. Hortic..

[47] Baslam M., Garmendia I., Goicoechea N. (2013). Enhanced Accumulation of Vitamins, Nutraceuticals and Minerals in Lettuces Associated with Arbuscular Mycorrhizal Fungi (AMF): A Question of Interest for Both Vegetables and Humans. Agriculture.

[48] Baslam M., Garmendia I., Goicoechea N. (2011). Arbuscular mycorrhizal fungi (AMF) improved growth and nutritional quality of greenhouse-grown lettuce. J. Agric. Food Chem..

[49] Kowalska I., Konieczny A. (2019). Effect of mycorrhiza on yield and quality of lettuce grown on medium with different levels of phosphorus and selenium. Agric. Food Sci..

[50] Rouphael Y., Cardarelli M., Bonini P., Colla G. (2017). Synergistic action of a microbial-based biostimulant and a plant derived-protein hydrolysate enhances lettuce tolerance to alkalinity and salinity. Front. Plant Sci..

[51] Chen S., Zhao H., Zou C., Li Y., Chen Y., Wang Z., Jiang Y., Liu A., Zhao P., Wang M. (2017). Combined Inoculation with Multiple Arbuscular Mycorrhizal Fungi Improves Growth, Nutrient Uptake and Photosynthesis in Cucumber Seedlings. Front. Microbiol..

[52] Al-Amri S.M., Elhindi K.M., El-Din A.F.S. (2016). Effects of arbuscular mycorrhizal fungus Glomus mosseae and phosphorus application on plant growth rate, essential oil content and composition of coriander (*Coriander sativum* L.). Prog. Nutr..

[53] Albrechtova J., Latr A., Nedorost L., Pokluda R., Posta K., Vosatka M. (2012). Dual inoculation with mycorrhizal and saprotrophic fungi applicable in sustainable cultivation improves the yield and nutritive value of onion. Sci. World J..

[54] Golubkina N., Amagova Z., Matsadze V., Zamana S., Tallarita A., Caruso G. (2020). Effects of Arbuscular Mycorrhizal Fungi on Yield, Biochemical Characteristics, and Elemental Composition of Garlic and Onion under Selenium Supply. Plants.

[55] Golubkina N., Zamana S., Seredin T., Poluboyarinov P., Sokolov S., Baranova H., Krivenkov L., Pietrantonio L., Caruso G. (2019). Effect of Selenium Biofortification and Beneficial Microorganism Inoculation on Yield, Quality and Antioxidant Properties of Shallot Bulbs. Plants.

[56] Castellanos-Morales V., Villegas J., Wendelin S., Vierheilig H., Eder R., Cárdenas-Navarro R. (2010). Root colonisation by the arbuscular mycorrhizal fungus Glomus intraradices alters the quality of strawberry fruits (Fragaria × ananassa Duch.) at different nitrogen levels. J. Sci. Food Agric..

[57] Mollavali M., Bolandnazar S.A., Schwarz D., Rohn S., Riehle P., Zaare Nahandi F. (2016). Flavonol Glucoside and Antioxidant Enzyme Biosynthesis Affected by Mycorrhizal Fungi in Various Cultivars of Onion (*Allium cepa* L.). J. Agric. Food Chem..

[58] Ahmad H., Hayat S., Ali M., Liu T., Cheng Z. (2018). The combination of arbuscular mycorrhizal fungi inoculation (Glomus versiforme) and 28-homobrassinolide spraying intervals improves growth by enhancing photosynthesis, nutrient absorption, and antioxidant system in cucumber (*Cucumis sativus* L.) under salinity. Ecol. Evol..

[59] Ganugi P., Martinelli E., Lucini L. (2021). Microbial biostimulants as a sustainable approach to improve the functional quality in plant-based foods: A review. Curr. Opin. Food Sci..

[60] Fernández I., Merlos M., López-Ráez J.A., Martínez-Medina A., Ferrol N., Azcón C., Bonfante P., Flors V., Pozo M.J. (2014). Defense Related Phytohormones Regulation in Arbuscular Mycorrhizal Symbioses Depends on the Partner Genotypes. J. Chem. Ecol..

[61] Cameron D.D., Neal A.L., van Wees S.C., Ton J. (2013). Mycorrhiza-induced resistance: More than the sum of its parts?. Trends Plant Sci..

[62] Giovannini L., Palla M., Agnolucci M., Avio L., Sbrana C., Turrini A., Giovannetti M. (2020). Arbuscular Mycorrhizal Fungi and Associated Microbiota as Plant Biostimulants: Research Strategies for the Selection of the Best Performing Inocula. Agronomy.

[63] Noceto P.A., Bettenfeld P., Boussageon R., Hériché M., Sportes A., van Tuinen D., Courty P.E., Wipf D. (2021). Arbuscular mycorrhizal fungi, a key symbiosis in the development of quality traits in crop production, alone or combined with plant growth-promoting bacteria. Mycorrhiza.

[64] Avio L., Maggini R., Ujvári G., Incrocci L., Giovannetti M., Turrini A. (2020). Phenolics content and antioxidant activity in the leaves of two artichoke cultivars are differentially affected by six mycorrhizal symbionts. Sci. Hortic..

[65] Ceccarelli N., Curadi M., Martelloni L., Sbrana C., Picciarelli P., Giovannetti M. (2010). Mycorrhizal colonization impacts on phenolic content and antioxidant properties of artichoke leaves and flower heads two years after field transplant. Plant Soil.

[66] Cardarelli M., Rouphael Y., De Pascale S., Bonini P., Colla G. Seed treatment with endophytic fungi enhances yield and nutritional quality of seed-propagated artichokes. Proceedings of the 10th International Symposium on Artichoke, Cardoon and Their Wild Relatives.

[67] Rouphael Y., Colla G., Graziani G., Ritieni A., Cardarelli M., De Pascale S. (2017). Phenolic composition, antioxidant activity and mineral profile in two seed-propagated artichoke cultivars as affected by microbial inoculants and planting time. Food Chem..

[68] Caser M., Victorino Í.M.M., Demasi S., Berruti A., Donno D., Lumini E., Bianciotto V., Scariot V. (2019). Saffron Cultivation in Marginal Alpine Environments: How AMF Inoculation Modulates Yield and Bioactive Compounds. Agronomy.

[69] Mollavali M., Perner H., Rohn S., Riehle P., Hanschen F.S., Schwarz D. (2018). Nitrogen form and mycorrhizal inoculation amount and timing affect flavonol biosynthesis in onion (*Allium cepa* L.). Mycorrhiza.

[70] Rozpądek P., Rąpała-Kozik M., Wężowicz K., Grandin A., Karlsson S., Ważny R., Anielska T., Turnau K. (2016). Arbuscular mycorrhiza improves yield and nutritional properties of onion (*Allium cepa*). Plant Physiol. Biochem..

[71] Avio L., Sbrana C., Giovannetti M., Frassinetti S. (2017). Arbuscular mycorrhizal fungi affect total phenolics content and antioxidant activity in leaves of oak leaf lettuce varieties. Sci. Hortic..

[72] Baslam M., Goicoechea N. (2012). Water deficit improved the capacity of arbuscular mycorrhizal fungi (AMF) for inducing the accumulation of antioxidant compounds in lettuce leaves. Mycorrhiza.

[73] Gholami R., Fahadi Hoveizeh N., Zahedi S.M., Gholami H., Carillo P. (2022). Melatonin alleviates the adverse effects of water stress in adult olive cultivars (*Olea europea* cv. Sevillana & Roughani) in field condition. Agric. Water Manag..

[74] González-González M.F., Ocampo-Alvarez H., Santacruz-Ruvalcaba F., Sánchez-Hernández C.V., Casarrubias-Castillo K., Becerril-Espinosa A., Castañeda-Nava J.J., Hernández-Herrera R.M. (2020). Physiological, Ecological, and Biochemical Implications in Tomato Plants of Two Plant Biostimulants: Arbuscular Mycorrhizal Fungi and Seaweed Extract. Front. Plant Sci..

[75] Salvioli A., Zouari I., Chalot M., Bonfante P. (2012). The arbuscular mycorrhizal status has an impact on the transcriptome profile and amino acid composition of tomato fruit. BMC Plant Biol..

[76] Kowalska I., Konieczny A., Gastol M., Sady W., Hanus-Fajerska E. (2015). Effect of mycorrhiza and phosphorus content in nutrient solution on the yield and nutritional status of tomato plants grown on rockwool or coconut coir. Agric. Food Sci..

[77] Bona E., Cantamessa S., Massa N., Manassero P., Marsano F., Copetta A., Lingua G., D’Agostino G., Gamalero E., Berta G. (2017). Arbuscular mycorrhizal fungi and plant growth-promoting pseudomonads improve yield, quality and nutritional value of tomato: A field study. Mycorrhiza.

[78] Schubert R., Werner S., Cirka H., Rödel P., Tandron Moya Y., Mock H.P., Hutter I., Kunze G., Hause B. (2020). Effects of Arbuscular Mycorrhization on Fruit Quality in Industrialized Tomato Production. Int. J. Mol. Sci..

[79] Nedorost L., Vojtíšková J., Pokluda R. (2014). Influence of watering regime and mycorrhizal inoculation on growth and nutrient uptake of pepper (*Capsicum annuum* L.). Acta Hortic..

[80] Avio L., Turrini A., Giovannetti M., Sbrana C. (2018). Designing the Ideotype Mycorrhizal Symbionts for the Production of Healthy Food. Front. Plant Sci..

[81] Agnihotri R., Sharma M.P., Prakash A., Ramesh A., Bhattacharjya S., Patra A.K., Manna M.C., Kurganova I., Kuzyakov Y. (2022). Glycoproteins of arbuscular mycorrhiza for soil carbon sequestration: Review of mechanisms and controls. Sci. Total Environ..

[82] Baslam M., Pascual I., Sánchez-Díaz M., Erro J., García-Mina J.M., Goicoechea N. (2011). Improvement of nutritional quality of greenhouse-grown lettuce by arbuscular mycorrhizal fungi is conditioned by the source of phosphorus nutrition. J. Agric. Food Chem..

[83] Baltazar M., Correia S., Guinan K.J., Sujeeth N., Bragança R., Gonçalves B. (2021). Recent advances in the molecular effects of biostimulants in plants: An overview. Biomolecules.

[84] Jimenez-Gomez A., Flores-Felix J.D., Garcia-Fraile P., Mateos P.F., Menendez E., Velazquez E., Rivas R. (2018). Probiotic activities of Rhizobium laguerreae on growth and quality of spinach. Sci. Rep..

[85] Khan A., Singh P., Srivastava A. (2018). Synthesis, nature and utility of universal iron chelator—Siderophore: A review. Microbiol. Res..

[86] Höfte M., Bakker P. (2007). Competition for Iron and Induced Systemic Resistance by Siderophores of Plant Growth Promoting Rhizobacteria. Microbial Siderophores.

[87] Romera F.J., García M.J., Lucena C., Martínez-Medina A., Aparicio M.A., Ramos J., Alcántara E., Angulo M., Pérez-Vicente R. (2019). Induced Systemic Resistance (ISR) and Fe Deficiency Responses in Dicot Plants. Front. Plant Sci..

[88] Lucena C., Romera F.J., García M.J., Alcántara E., Pérez-Vicente R. (2015). Ethylene Participates in the Regulation of Fe Deficiency Responses in Strategy I Plants and in Rice. Front. Plant Sci..

[89] Camelo M., Vera S.P., Bonilla R.R. (2011). Mechanisms of Action of Plant Growth Promoting Rhizobacteria. Cienc. Y Tecnol. Agropecu..

[90] García-Fraile P., Carro L., Robledo M., Ramírez-Bahena M.H., Flores-Félix J.D., Fernández M.T., Mateos P.F., Rivas R., Igual J.M., Martínez-Molina E. (2012). Rhizobium promotes non-legumes growth and quality in several production steps: Towards a biofertilization of edible raw vegetables healthy for humans. PLoS ONE.

[91] Ayuso-Calles M., Garcia-Estevez I., Jimenez-Gomez A., Flores-Felix J.D., Escribano-Bailon M.T., Rivas R. (2020). Rhizobium laguerreae Improves Productivity and Phenolic Compound Content of Lettuce (*Lactuca sativa* L.) under Saline Stress Conditions. Foods.

[92] Jiménez-Gómez A., García-Estévez I., Escribano-Bailón M.T., García-Fraile P., Rivas R. (2021). Bacterial Fertilizers Based on Rhizobium laguerreae and Bacillus halotolerans Enhance Cichorium endivia L. Phenolic Compound and Mineral Contents and Plant Development. Foods.

[93] O’Callaghan M., Ballard R.A., Wright D. (2022). Soil microbial inoculants for sustainable agriculture: Limitations and opportunities. Soil Use Manag..

[94] Cassán F., Coniglio A., López G., Molina R., Nievas S., de Carlan C.L.N., Donadio F., Torres D., Rosas S., Pedrosa F.O. (2020). Everything you must know about Azospirillum and its impact on agriculture and beyond. Biol. Fertil. Soils.

[95] Xu Z., Pehlivan N., Ghorbani A., Wu C. (2022). Effects of Azorhizobium caulinodans and Piriformospora indica Co-Inoculation on Growth and Fruit Quality of Tomato (*Solanum lycopersicum* L.) under Salt Stress. Horticulturae.

[96] Chanratana M., Joe M.M., Roy Choudhury A., Anandham R., Krishnamoorthy R., Kim K., Jeon S., Choi J., Choi J., Sa T. (2019). Physiological response of tomato plant to chitosan-immobilized aggregated Methylobacterium oryzae CBMB20 inoculation under salinity stress. 3 Biotech.

[97] Pérez-Rodriguez M.M., Piccoli P., Anzuay M.S., Baraldi R., Neri L., Taurian T., Lobato Ureche M.A., Segura D.M., Cohen A.C. (2020). Native bacteria isolated from roots and rhizosphere of *Solanum lycopersicum* L. increase tomato seedling growth under a reduced fertilization regime. Sci. Rep..

[98] Galleguillos C., Aguirre C., Miguel Barea J., Azcón R. (2000). Growth promoting effect of two Sinorhizobium meliloti strains (a wild type and its genetically modified derivative) on a non-legume plant species in specific interaction with two arbuscular mycorrhizal fungi. Plant Sci..

[99] Young J.M. (2003). The genus name Ensifer Casida 1982 takes priority over Sinorhizobium Chen et al. 1988, and Sinorhizobium morelense Wang et al. 2002 is a later synonym of Ensifer adhaerens Casida 1982. Is the combination ‘Sinorhizobium adhaerens’ (Casida 1982) Willems et al. 2003 legitimate? Request for an Opinion. Int. J. Syst. Evol. Microbiol..

[100] Didonato Floro R., Lee J., Bogosian G., Bryant D. (2014). Compositions and methods for improving tomato production. U.S. Patent.

[101] Fasciglione G., Casanovas E.M., Yommi A., Sueldo R.J., Barassi C.A. (2012). Azospirillum improves lettuce growth and transplant under saline conditions. J. Sci. Food Agric..

[102] Kopta T., Pavlikova M., Sekara A., Pokluda R., Marsalek B. (2018). Effect of Bacterial-algal Biostimulant on the Yield and Internal Quality of Lettuce (*Lactuca sativa* L.) Produced for Spring and Summer Crop. Not. Bot. Horti Agrobot. Cluj-Napoca.

[103] Kopta T., Pokluda R., Marsalek B. (2016). Effect of algae and bacteria application on nutritional value of selected leafy vegetables. Acta Hortic..

[104] Kolega S., Miras-Moreno B., Buffagni V., Lucini L., Valentinuzzi F., Maver M., Mimmo T., Trevisan M., Pii Y., Cesco S. (2020). Nutraceutical Profiles of Two Hydroponically Grown Sweet Basil Cultivars as Affected by the Composition of the Nutrient Solution and the Inoculation With Azospirillum brasilense. Front. Plant Sci..

[105] Kordi S., Salmasi S.Z., Kolvanagh J.S., Weisany W., Shannon D.A. (2020). Intercropping System and N-2 Fixing Bacteria Can Increase Land Use Efficiency and Improve the Essential Oil Quantity and Quality of Sweet Basil (*Ocimum basilicum* L.). Front. Plant Sci..

[106] Oancea F., Raut I., Zamfiropol Cristea V. (2017). Influence of soil treatment with microbial plant biostimulant on tomato yield and quality. Agric. Food.

[107] El-Beltagi H.S., Ahmad I., Basit A., Abd El-Lateef H.M., Yasir M., Shah S.T., Ullah I., Mohamed M.E.M., Ali I., Ali F. (2022). Effect of Azospirillum and Azotobacter Species on the Performance of Cherry Tomato under Different Salinity Levels. Gesunde Pflanz..

[108] Pérez-Velasco E., Mendoza-Villarreal R., Sandoval Rangel A., De la Fuente M., Robledo-Torres V., Valdez-Aguilar L. (2019). Evaluación del uso de endomicorrizas y Azospirillum sp. en la productividad y calidad nutracéutica de chile morrón (Capsicum annuum) en invernadero. Inf. Técnica Económica Agrar..

[109] Mirshekari B., Valizadeh N., Roudsari A.M., Maleki S.H., Farahvash F., Kouchebagh S.B. (2010). Improved growth and essential oil quality of Foeniculum vulgare by Azospirillum inoculation and nutrient seed priming. J. Food Agric. Environ..

[110] Jimenez-Gomez A., Garcia-Estevez I., Garcia-Fraile P., Escribano-Bailon M.T., Rivas R. (2020). Increase in phenolic compounds of Coriandrum sativum L. after the application of a Bacillus halotolerans biofertilizer. J. Sci. Food Agric..

[111] Toledo Cabrera B. (2021). Effect of Rhizobium Inoculation on Tomato (*Solanum lycopersicum* L.) Yield in Protected Crops. Biol. Life Sci. Forum.

[112] Giovannetti M., Avio L., Sbrana C., Ramawat K., Mérillon J.M. (2013). Improvement of nutraceutical value of food by plant symbionts. Natural Products: Phytochemistry, Botany and Metabolism of Alkaloids, Phenolics and Terpenes.

[113] Agnolucci M., Avio L., Palla M., Sbrana C., Turrini A., Giovannetti M. (2020). Health-promoting properties of plant products: The role of mycorrhizal fungi and associated bacteria. Agronomy.

